# Comaneci device-assisted embolization of wide-necked carotid aneurysms with an unfavorable ratio

**DOI:** 10.1186/s12883-020-01963-2

**Published:** 2020-10-22

**Authors:** Juan David Molina-Nuevo, Lorena López-Martínez, María José Pedrosa-Jiménez, Enrique Juliá-Molla, Francisco Hernández-Fernández

**Affiliations:** 1 Radiology department, Complejo Hospitalario Universitario Albacete, Albacete, Spain; 2 C. Hospitalario Universitario de Albacete, C. Hermanos Falcó nº 37. CP, 02006 Albacete, Spain; 3 Neurology department, Complejo Hospitalario Universitario Albacete, Albacete, Spain

**Keywords:** Aneurysm, Comaneci, Remodeling, Embolization

## Abstract

**Background:**

Endovascular treatment is the technique of choice for most intracranial aneurysms. However, the treatment of morphologically complex wide-necked aneurysms with an unfavorable anatomy is still a therapeutic challenge.

The purpose of the study is to describe the initial experience with the Comaneci embolization assist device for the treatment of wide-necked aneurysms with an unfavorable ratio for direct embolization.

**Methods:**

We report a retrospective single-center analysis taken from a prospective database of consecutive aneurysms of the anterior circulation treated using the *Comaneci* device in the period from March 2017 to March 2019.

**Results:**

Eighteen aneurysms were collected from 16 patients (9 women and 7 men) treated using the *Comaneci* device. The mean age was 48.4 years (range 36–81). Twelve patients had SAH, three were incidental aneurysms and one had compressive symptoms. A complete asymptomatic occlusion rate of 88.8% was obtained. The major complication rate was 5.55%.

**Conclusion:**

The Comaneci embolization assist device is a safe, effective option for endovascular treatment of complex aneurysms with an unfavorable ratio.

## Background

Endovascular treatment is the technique of choice for most intracranial aneurysms [1, 2]. In cases with a complex or unfavorable anatomy, additional devices may be needed in order to achieve a complete and safe treatment of the aneurysm. Balloon-assisted coiling (BAC) involves placement of a removable compliant balloon adjacent to the aneurysm neck. Stent-assisted coiling involves placement of a permanent stent that covers the neck of the aneurysm. Both techniques provide a scaffold that improves aneurysm neck coverage and at the same time prevents coil herniation into the parent vessel [3].

In this context, the Comaneci temporary embolization assist device (*Rapid Medical*, Yokneam, Israel) has appeared as a new tool for the treatment of intracranial aneurysms with an unfavorable anatomy.

The main characteristics of this device include the ability to cover temporally the aneurysm neck allowing safe embolization using coils, without needing to administer antiaggregant treatment in the long term (unlike SAC) and the ability to keep blood flow patent through the device during the treatment (unlike BAC) [4–7].

The primary objective of this study is to report our initial experience in the treatment of ruptured or incidental aneurysms of the anterior circulation using the Comaneci temporary embolization assist device.

## Methods

The Comaneci temporary embolization assist device is a stent comprising 12 nitinol wire bundles assembled over a central bundle of 182 cm that shows a flexible tip in its most distal portion (last 7 mm) in order to improve navigability. Its entire structure is visible as it is made of nitinol. In its most proximal portion it shows the release control, which allows to expand the device to reach the desired caliber, and thus obtain complete apposition to the vascular wall, cover the aneurysm neck reliably and improve the dome-neck ratio (aspect ratio). In this way an adequate retention and compaction of the coils in the aneurysm sac may be obtained.

**Fig. 1:**
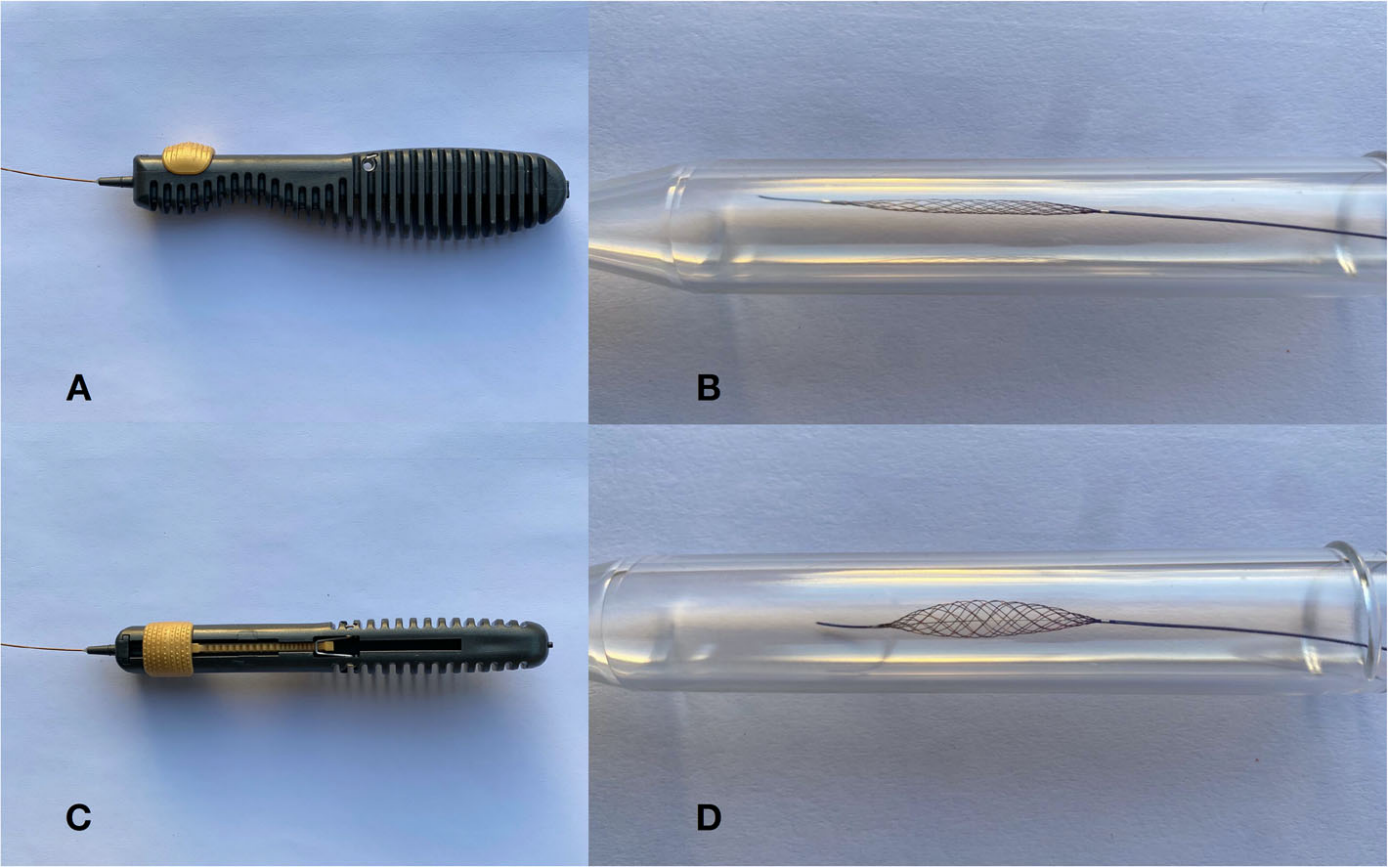
**a** and **c**: Detail of the Comaneci device handle. Expansion and contraction are achieved by pulling or pushing, respectively, the golden slider. **b**: Contracted Comaneci device. **d:** Expanded Comaneci device

There are three versions. The standard model, *Comaneci*, has a length of 35 mm and reaches a width of up to 4.5 mm when expanded (Fig. 1). This device is compatible with 0.021″ microcatheters. The intermediate version, *Comaneci Petit*, has a length of 24 mm and is expanded to reach a width of 3.5 mm, and is also compatible with 0.021″ microcatheters. Finally, *Comaneci 17* has a length of 17 mm, a width of up to 3 mm and is compatible with 0.017″ microcatheters.

All versions have CE (*Conformité Européenne*) and FDA (Food and Drug Administration) marking.

The study reported herein is a single-center, retrospective analysis taken from a prospective database - previously approved by our institution’s ethics committee - of consecutive aneurysms of the anterior circulation treated using the *Comaneci* device in the period from March 2017 to March 2019.

Patients with incidental, compressive and ruptured aneurysms were included and evaluated by a multidisciplinary team comprising interventional neuroradiologists, neurosurgeons, neurologists and intensivists. Patients with subarachnoid hemorrhage (SAH) were admitted to a critical care unit and their scores in the Glasgow, Fisher and Hunt-Hess scales were recorded. The decision to perform treatment using a Comaneci assist device was made by the team of interventional neuroradiologists and neurologists in charge of performing the embolization treatment based on the morphological characteristics, measures, aspect ratio and location of the aneurysm.

The morphological characteristics, maximum diameter, aspect ratio, location, type of *Comaneci* device used and the final outcome were collected. All cases were treated by interventional neuroradiologists and neurologists with over 5 years of experience, considering as first indication for the use of the Comaneci device the presence of a wide-neck aneurysm (greater than 4 mm) and/or configuration of the aneurysm sac unfavorable for direct embolization (aspect ratio limit: 2). Written informed consent was obtained from every patient or direct relatives before each treatment according to our institutional policies related to ethical issues.

### Endovascular procedures

All procedures were performed under general anesthesia. Prior to the embolization, a central venous catheter was placed and a radial artery was canalized for continuous blood pressure (BP) monitoring. An Innova GE monoplane angiograph (*General Electric Company*, Boston, MA, US) was used.

Transfemoral arterial access was obtained, a 6 or 8F introducer was implanted and a diagnostic angiography was performed, that included three-dimensional (3D) rotational angiography. The images obtained were processed in a GE *Workstation* 4.6, and the morphology of the sac, measures of the aneurysm and neck length were evaluated to assess the need for embolization assist techniques.

After the end of the diagnostic tests, diagnostic catheter exchange was performed using a 0.035″ guidewire of 260 cm (*Terumo Corporation,* Shibuya, Tokyo, Japan) and a Neuron MAX 0.88 guide catheter (*Penumbra Inc*., Alameda, CA, US) was implanted in all cases. Reconstructions were made to identify an adequate work projection and navigation was performed by fluoroscopy and road mapping.

**Fig. 2:**
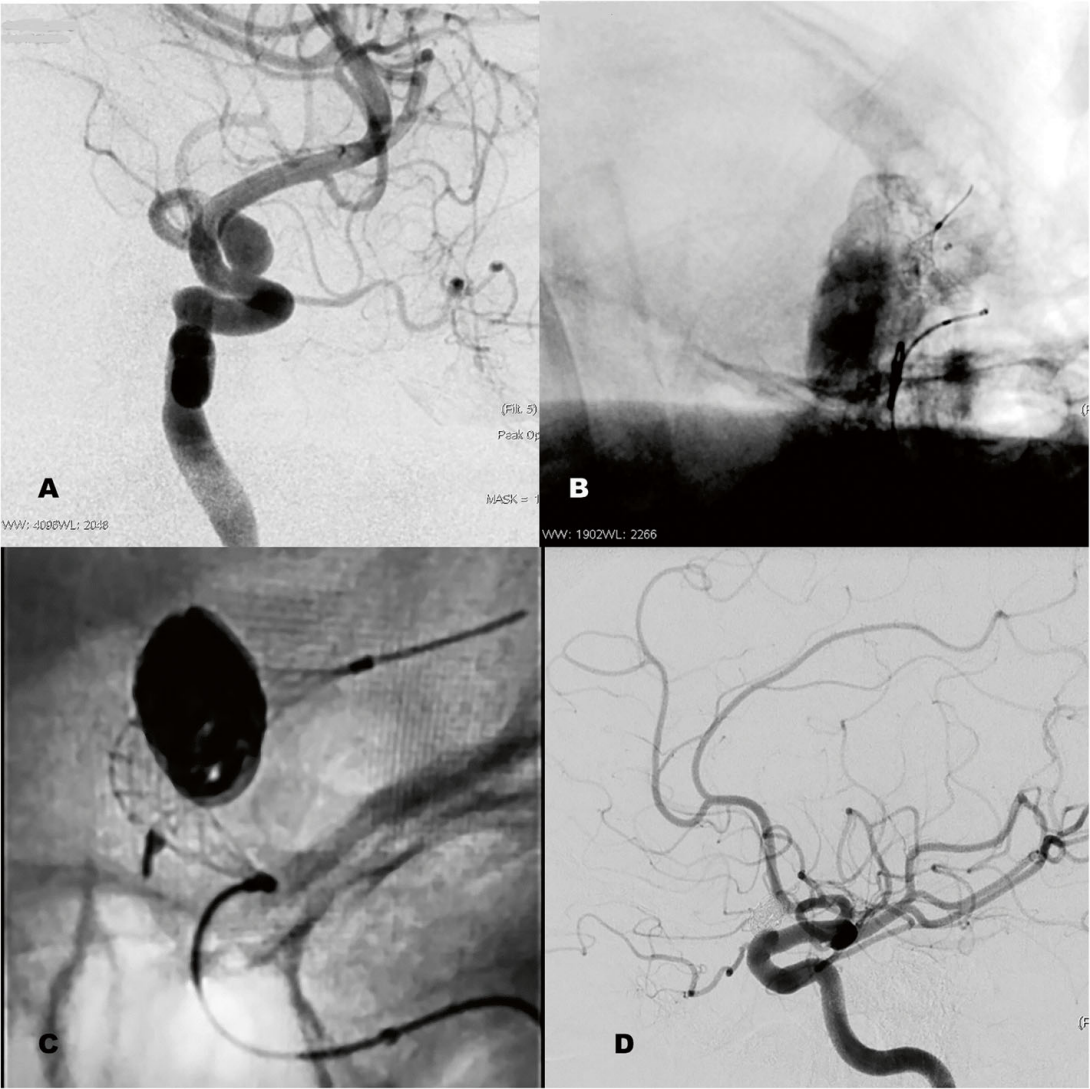
Comaneci embolization assist device. **a**: Aneurysm of paraophthalmic segment carotid. **b**: Deployment of the Comaneci device with embolization microcatheter in the aneurysm sac. Jailing technique. **c**: Complete aneurysm embolization. **d**: Arteriographic control one year later

A microcatheter carrying the *Comaneci* device was first placed, with its terminal edge distal to the neck of the aneurysm, anticipating the shortening of the Comaneci during its deployment, thus achieving adequate release of the device and complete coverage of the aneurysm neck. The selection of the navigation microcatheter for *Comaneci* depends on the device selected. For *Comaneci* and *Comaneci Petit* a Rebar 18 microcatheter (*Medtronic plc*, Dublin, Ireland) was used and for *Comaneci 17* an Echelon 10 microcatheter (*Medtronic plc*, Dublin, Ireland) was used. Once the microcatheter carrying the Comaneci device reached the desired position, the embolization microcatheter was navigated into the aneurysm sac. A 45° curved Echelon 10 microcatheter (*Medtronic plc*, Dublin, Ireland) was used in all cases. Navigation of both the Comaneci device carrier microcatheter and the embolization microcatheter was assisted by a Traxcess 0.014″ hydrophilic microguide (*Microvention Inc*., Aliso Viejo, CA, US). Once both microcatheters were in place, the Comaneci device was deployed bridging the aneurysm neck and fixing the embolization microcatheter inside the aneurysm sac. This process was carried out in a slow and controlled manner, obtaining multiple projections to ensure complete coverage of the aneurysm neck, proper artery wall apposition and also the correct position of the distal microcatheter tip within the aneurysm sac (Fig. 2). The authors recommend slow opening of the device, to thus minimize the risk of uncontrolled movements of the microcatheter tip within the aneurysm sac. Avoiding over-dilation of the Comaneci device should also be considered at this point, since, according to the authors’ experience, this promotes vasospasm and the appearance of platelet aggregates. Thanks to the new control grip of the device that provides a staggered deployment with automatic fixing, preventing the device from folding when the pressure ceases, and which does not require activation of any fixing device, this process can be carried out in a relatively easy and controlled way with a single hand which greatly facilitates the procedure.

Subsequently, the coils were released into the aneurysm sac to obtain complete embolization. At the end of the embolization phase, the *Comaneci* device was slowly folded (contracted), without changing its position, checking the stability of the coil mesh inside the aneurysm sac. This process must be carried out with extreme care and very slowly, avoiding an abrupt folding of the device that could destabilize the mesh of coils and checking at all times the absence of coil entanglement with the Comaneci device.

The embolization microcatheter was withdrawn from the aneurysm sac after the Comaneci device folding. Finally, after retrieval of the embolization microcatheter, the Comaneci device was re-sheathed in its navigation microcatheter, and both were removed.

### Intraprocedure drugs

**Fig. 3:**
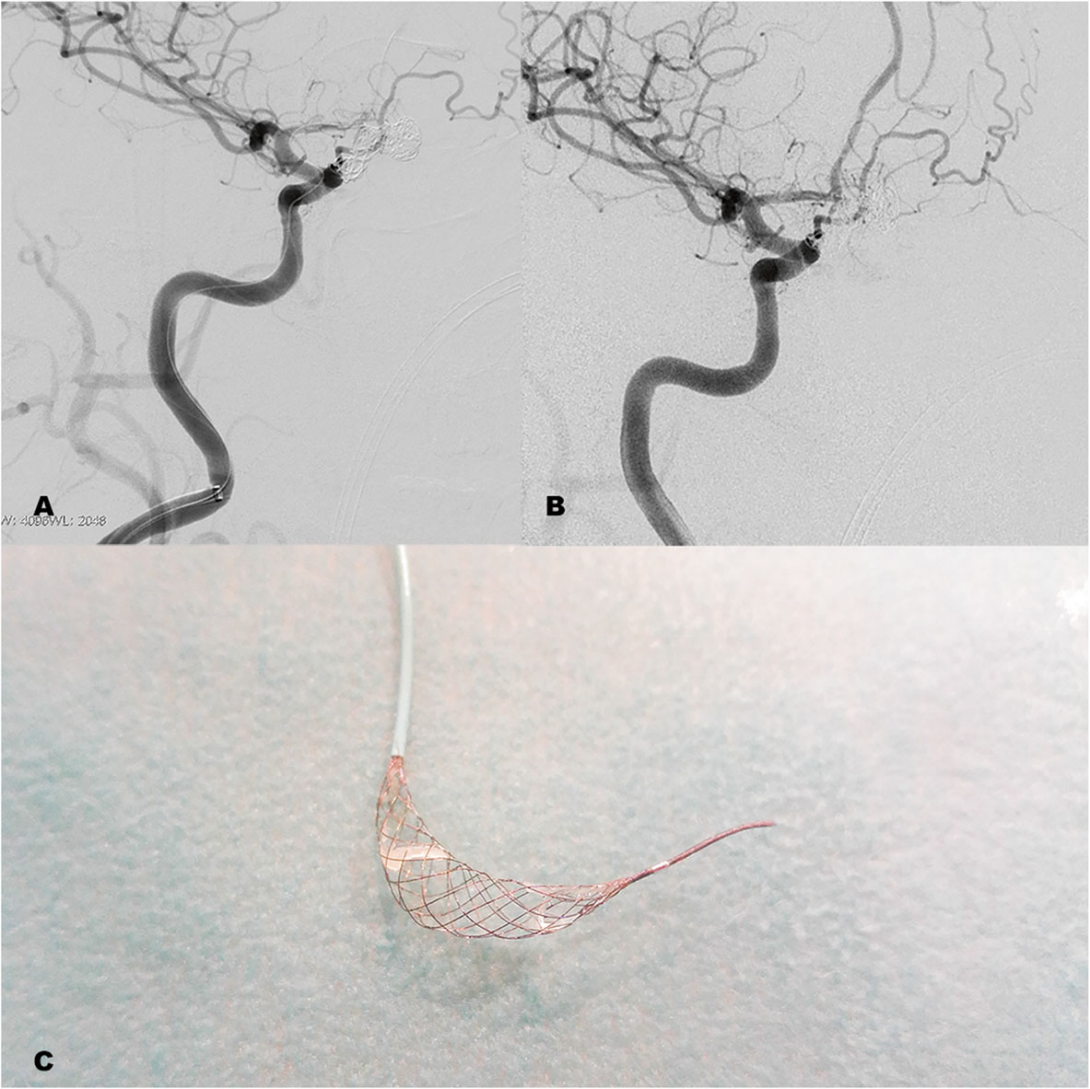
Embolization of complex AcoA aneurysm assisted by Comaneci 17. **a**: Complete aneurysm embolization and pseudoocclusion of RACA by platelet aggregates. **b**: Arteriographic control 10 min after rapid withdrawal of the Comaneci device and increased blood pressure. **c**: Detail of the Comaneci device with presence of macroscopic platelet aggregates

Patients with incidental aneurysms received premedication with clopidogrel 75 mg and acetyl salicylic acid (ASA) 100 mg from 5 days before the procedure. In the rest of the cases no periprocedure antithrombotic drug was indicated. In case platelet aggregates were seen, with or without slowing down of arterial flow during embolization (Fig. 3), an intravenous loading dose of ASA 450 mg was administered and blood pressure (BP) was increased with vasoactive drugs (ephedrine). Furthermore, in case placement of the coils had ended, the *Comaneci* device was withdrawn as soon as possible.

### Follow-up

In all cases a computerized tomography (CT) was performed at 24 h of the treatment to rule out post-procedure complications. Patients were followed-up at the Neurology outpatient clinic by a member of our neurointerventional team, at 3 months of hospital discharge and the mRs (*modified Rankin scale*) was recorded. Angiographic follow-up was performed at 3, 6 months and subsequently at 1 year and at 2 years. Angiographic outcomes were classified based on the modified Raymond-Roy classification [8].

### Complications

Major complications were defined as aneurysm rupture or any other type of intracranial bleeding and final ischemic lesion, either in the territory of the vessel to be treated (local) or in any other territory (at distance). The appearance of endovascular aggregates, reversible vasospasm and complications of the vascular access were defined as minor complications.

## Results

Eighteen aneurysms were treated in 16 patients (9 women and 7 men) using the *Comaneci* device, as shown in Table 1. Cases 15 and 16 as well as 17, 18 represent the treatment of “mirror” carotid aneurysms in two different patients. The mean age was 48.4 years (range 36–81). Embolized aneurysms were located in the anterior communicating artery (ACoA), [2] posterior communicating artery (PComA), [8] paraophthalmic segment of the internal carotid artery (PophCA) [4] and terminal internal carotid artery (TICA) [4]. Two of the cases treated were retreatments of previously embolized aneurysms: one of them assisted with compliant balloon (case 1) and another due to aneurysm regrowth in association with dysplasia of ACoA and previous embolization with coils (case 4).

**Table 1:** PophCA (Paraophthalmic Internal Carotid Artery), TICA (Terminal Internal Carotid Artery), ACoA (Anterior Communicating Artery), PComA (Posterior Communicating Artery), SAH (subarachnoid hemorrhage), Sac (Saccular), 3 m mRS (3-month modified Rankin scale)

Case No.	Site	Type/ Max. diameter/ Aspect ratio	***Comaneci version***	Clinical manifestation	Result mod. Raymond-Roy Class.	Complications	3 m mRs
1	PComA	Sac/ 8 mm/ 2	*Comaneci*	SAH	2	NO	0
2	PophCA	Sac/ 8 mm/ 2	*Comaneci*	Incidental	1	NO	0
3	TICA	Sac/ 7 mm/ 1.60	*Comaneci*	SAH	1	NO	0
4	ACoA	Sac/ 6 mm/ 1	*Comaneci 17*	SAH	1	Platelet aggregates without ischemia	6
5	PophCA	Sac/ 13 mm/ 4.30	*Comaneci*	Compressive symptoms	1	Anterior choroidal artery stroke	1
6	PophCA	Sac/ 4 mm/ 2	*Comaneci*	Incidental	1	NO	0
7	TICA	Sac/ 6 mm/ 1.3	*Comaneci*	SAH	1	Platelet aggregates without ischemia	1
8	TICA	Fusiform/ 6 mm	*Comaneci*	SAH	–	NO	2
9	PophCA	Sac/ 8 mm/ 2	*Comaneci*	Incidental	1	Coil migration (retrieved)	0
10	TICA	Sac/ 8 mm/ 2.60	*Comaneci*	SAH	1	NO	6
11	PComA	Sac/ 3 mm/ 1.50	*Comaneci*	SAH	1	NO	0
12	PComA	Sac/ 5 mm/ 1.30	*Comaneci 17*	SAH	1	NO	0
13	PComA	Sac/ 8 mm/ 1.6	*Comaneci*	SAH	1	NO	0
14	ACoA	Sac/ 9 mm/ 1.70	*Comaneci 17*	SAH	1	NO	4
15	PComA	Sac/ 2.5 mm/ 0.83	*Comaneci* *Petit*	SAH	1	NO	2
16	PComA	Sac/ 2.6 mm/ 1	*Comaneci* *Petit*	SAH	1	NO	2
17	PComA	Sac/ 4.5 mm/ 1.5	*Comaneci*	SAH	1	NO	0
18	PComA	Sac/ 4.5 mm/ 1.2	*Comaneci*	SAH	1	NO	0

The aneurysms treated had a mean diameter of 6.02 mm (range 2.5 to 13). Three cases had incidental aneurysms, one with compressive symptoms (non-ruptured) and 14 ruptured aneurysms. These cases occurred as subarachnoid hemorrhage (SAH), with Fisher grade 4 in 11 cases and Fisher grade 3 in two.

Technical success, defined as satisfactory embolization (Raymond-Roy Class I-II) of the aneurysm to be treated, was obtained in all cases except one (case 8), probably due to a wrong choice of the device, as it was a ruptured fusiform carotid aneurysm. Urgent treatment was attempted in this case, but adequate apposition of the device to the ill vessel or coil disposition stability were not achieved, so the treatment was dismissed. Finally, this case was treated in a second time using a flow diverter stent.

Complete embolization of the aneurysm was achieved in all cases except one (case 1), where a stable neck remnant of 2 mm persisted at two-year follow-up.

The mean angiographic follow-up of our cases was 9.3 months (range 0–24). No angiographic follow-up was performed in cases 4, 5, 8, 15 and 16, the first due to death of the patient, the second due to refusal to the arteriography and the other due to referral to the reference site.

Only one case of aneurysm regrowth was detected in our Comaneci device assisted embolization aneurysm series. It was case 5, which presented a new episode of SAH 2 years after the first embolization, and was re-treated using a flow diverter. Complications were registered in four cases in our series (Table 1). Three minor complications occurred: appearance of platelet aggregates in two cases (cases 4 and 7), which was solved during the procedure, and one case of coil migration (case 9), considered a minor complication as it could be solved satisfactorily using the *Comaneci device as extractor, and the coil could be withdrawn* without causing any ischemic or hemorrhagic lesion. With regard to the platelet aggregates produced (cases 4 and 7) they could be solved with antiaggregant agents (loading dose of ASA), BP increase or withdrawal of the device. A major complication was registered (case 5): ischemic stroke in the territory of the anterior choroidal artery (AChoA) after embolization of a giant aneurysm in the PComA. The subsequent analysis of case 5 did not show distortion of the coil mesh or occlusion of the AChoA due to coil protrusion, so inadvertent partial occlusion of the origin of the AChoA by *Comaneci* filaments during deployment, distortion of the flow caused by the device or compressive events on the AChoA derived from embolization itself should probably be considered the cause of ischemia. This patient was treated with ASA 100 mg once the infarction was demonstrated, and his motor deficit progressed favorably and with very few sequels (mRs at 3 months = 1).

## Discussion

### Key results

Although embolization is currently the treatment of choice for most intracranial aneurysms, [1, 2] those with an unfavorable ratio, giant aneurysms or those presenting unfavorable morphologies remain a therapeutic challenge, significantly increasing the complications rate in these cases [9].

In this article we report a series of 18 aneurysms with unfavorable ratios that were embolized assisted by the Comaneci device, achieving technical success (Raymond Class I-II) in all cases except 1 (94.4%) and with only one major complication (5.5%). These data are comparable to those published in the scientific literature [1, 2, 9] and to those obtained in our general aneurysm embolization series. We also provide a complete intraprocedure drug administration protocol and recommendations for the event of complications during Comaneci device-assisted embolization.

### Interpretations

There are well-known embolization strategies that allow a safe and effective treatment of aneurysms with an unfavorable ratio. The most representative examples are compliant balloon-assisted coiling (BAC) and stent-assisted coiling (SAC). Both are based on temporary (BAC) or permanent (SAC) creation of an aneurysm neck coverage that supports the coil mesh, enhancing retention inside the aneurysm sac and promoting effective embolization [9, 10]. Embolization using flow diverter stents is an alternative to the previous two techniques, [9, 11, 12] as well as embolization using intravascular devices [13]. Finally, vascular sacrifice is still considered a valid therapeutic option for this condition [14].

BAC consists of temporary coating of the aneurysm neck with a compliant balloon that is inflated and facilitates compaction and retention of the coil mesh inside the aneurysm sac. The main advantages include no need for additional antithrombotic treatment and the ability to control bleeding in case of eventual rupture of the aneurysmal sac during embolization. The inconveniences of this technique are the disruption of the intracranial blood flow with each balloon inflation, the difficulty for the compliant balloon to navigate in very tortuous arteries and the lack of stability and possible coil mesh migration after deflation. In addition, the repetition of compliant balloon inflation or deflation may be associated with the appearance of thromboembolic events, endothelial damage or vasospasm [3, 15].

SAC arises in response to the main inconveniences of BAC as it does not cause intracranial flow disruption, it may be a valid alternative in very tortuous anatomies and provides a permanent support over the aneurysm neck that will prevent migration of the coil mesh at all times. In contrast, it involves the need for additional drugs (dual antiplatelet therapy), which makes its use controversial in the case of ruptured aneurysms [16, 17]. The same problem occurs in the treatment with flow diverter stents, since, as in the case of SAC, the use of permanent antiplatelet therapy is mandatory [18].

The new concept of “temporary device-assisted coiling” arises in this context attempting to combine the advantages and excluding the disadvantages of BAC and SAC. The application of devices that provide effective coverage of the aneurysm without disrupting intracranial flow and without needing repeated inflation can reduce the possibility of thromboembolic events and endothelial damage. In addition, the possibility of removing the device once embolization is completed makes permanent dual antiplatelet therapy unnecessary. Comaneci was the first device developed according to the new concept of “temporary device-assisted coiling” presenting all the features previously described. The new Cascade device (Perflow Medical, Netanya Israel) has been recently presented as an alternative to the Comaneci device. Although both devices have a very similar mechanism of action, providing a temporal neck bridging [19], there are significant differences between them. Thus, the Cascade device has a tighter mesh than the Comaneci, but it is only compatible with 0.021″ microcatheters, while the Comaneci “17” version is compatible with 0.017″ microcatheters, and can be used in very small arteries or in highly tortuous vascular territories. Further studies comparing the efficacy of both devices will be needed.

There are multiple articles in the scientific literature concluding that the use of the Comaneci device is safe and provides adequate occlusion rates in aneurysm embolization [20]. The results of our series are consistent with these other published series and support the usefulness of the *Comaneci* embolization assist device for the treatment of complex aneurysms, confirming that this device is a real alternative to BAC and SAC. To be noted is the complete occlusion rate reached, 16/18 cases (88.8%), higher than that of the direct embolization technique [1, 2] and that shown in several published series of BAC or SAC [3, 21]. Furthermore, only one aneurysm has undergone recanalization (5.55%) since the current literature reports percentages of up to 20% in the recanalization rate of carotid aneurysms [22].

**Fig. 4:**
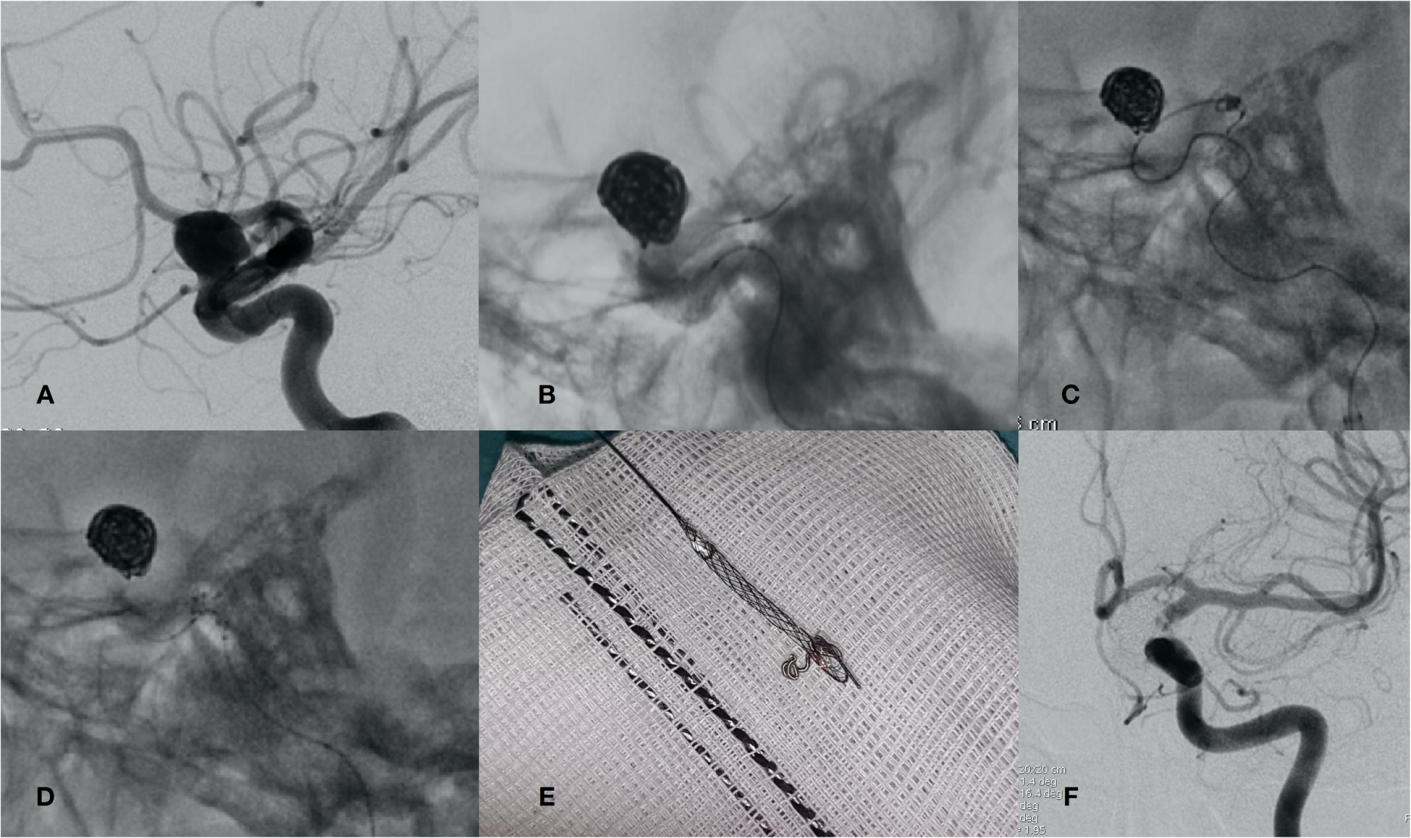
**a**: Paraophthalmic aneurysm with unfavorable ratio. **b**: Completion of assisted embolization using the Comaneci device. **c**: Deployment of the Comaneci device at the height of the migrated coil. **d**: Removal of the migrated coil in conjunction with the Comaneci device. **d**: Detail of the coil captured by the Comaneci device. **e**: Angiographic control 6 months after treatment showing Raymond-Roy classification Grade I embolization

However, while the Comaneci device shows promising results and offers significant advantages over BAC and SAC, it also has some drawbacks. Thus, the Comaneci device cannot control bleeding in the event of aneurysm rupture, which can occur in balloon-assisted embolization. Also, although being a removable device involves a significant advantage over the use of stents, its withdrawal is still a risky maneuver in which a coil can detach from the rest of the mesh or become entangled with the device. In this regard, our study includes one case (case 9) in which a coil detached from the intrasaccular mesh during the Comaneci withdrawal maneuver, and migrated distally. The complication was most likely caused by entanglement of the coil that migrated over the *Comaneci’s* filaments. This went unnoticed and caused the *coil* to be pulled to disengage from the intrasaccular mesh during folding of the Comaneci device. After noticing distal migration of the coil, we advanced the *Comaneci* and deployed it again at the height of the coil migrated, thus being able to trap and withdraw it without clinical consequences (Fig. 4).

Finally, in this study we also establish a premedication regimen for both ruptured and unruptured aneurysms. In addition, we provide precise technical solutions for the hypothetical case of complications. In this regard, our series shows that the appearance of platelet aggregates is a more common complication than reported in previous publications [5, 6, 20]. The appearance of these aggregates may be related to the time during which the *Comaneci* device remains deployed in a hypercoagulability scenario. Further studies will be required to establish the actual incidence of this complication in aneurysm embolization using the *Comaneci* device and the potential need for periprocedure antiaggregants.

### Limitations and generalizability

The study shows some limitations, first methodological. It is a single-center, observational, retrospective study that analyzes a relatively small sample of 18 cases. In addition, although the follow-up time exceeds 1 year in all but one case (Case 9: 6 months) in five patients no angiographic follow-up was performed for the reasons previously explained. The method for resolution of thromboembolic complications has been at the discretion of the interventional physician, not following a pre-established protocol. The series included only patients with anterior circulation aneurysms as during this period there were no posterior circulation aneurysms that met the anatomic characteristics for selecting the *Comaneci* device.

## Conclusion

Our initial results in the assisted embolization of complex aneurysms with the *Comaneci device s*how a high complete occlusion rate in ruptured or incidental anterior circulation aneurysms, with a low rate of major complications and no recanalization. Based on the data provided, we consider that the Comaneci aneurysm embolization assist device is a safe, effective option for endovascular treatment of complex carotid aneurysms with an unfavorable ratio.

## Data Availability

The datasets generated and analysed during the current study are not publicly available due to privacy reasons of patients, but are available from the corresponding author on reasonable request.

## Abbreviations

CE: Conformité Européenne
FDA: Food and drug administration
SAH: Subarachnoid hemorrhage
BP: Blood pressure
GE: General electric company
ASA: Acetyl salicylic acid
CT: Computed tomography
mRs: Modified rankin scale
ACoA: Anterior communicating artery
AChoA: Anterior choroidal artery
PComA: Posterior communicating artery
PophCA: Paraophthalmic internal carotid artery
TICA: Terminal internal carotid artery
BAC: Balloon assisted coiling
SAC: Stent assisted coiling
